# Quantification of Single‐Cell Cysteine Using an Electrochemical Nanosensor for Predicting Tumor Disulfidptosis Susceptibility

**DOI:** 10.1002/advs.202523478

**Published:** 2025-12-22

**Authors:** Congcong Zhang, Xiangdi Zhang, Shushen Li, Yuyang Li, Bei Yuan, Mingshuang Zheng, Shuo Zhang, Fangping Yuan, Min Jia, Lixia Lu, Jun Zhou, Zhenguo Zhang, Xin Du

**Affiliations:** ^1^ Center for Cell Structure and Function Shandong Provincial Key Laboratory of Animal Resistance Biology College of Life Sciences Shandong Normal University Jinan Shandong China; ^2^ Department of Clinical Pathobiology and Immunological Testing College of Medical Laboratory Qilu Medical University Zibo Shandong China

**Keywords:** cysteine, disulfidptosis, electrochemical, nanosensor, single‐cell

## Abstract

Disulfidptosis, a newly identified form of programmed cell death, has emerged as a promising therapeutic target for tumors. However, the current research in this field is hindered by the absence of precise and quantifiable biomarkers to accurately predict and monitor its occurrence and progression. Research has demonstrated a strong correlation between disulfidptosis and cysteine metabolism. To elucidate the relationship, this study pioneers the development of a novel carbon fiber nanoelectrode (CFNE) enhanced with platinum nanoparticles and poly(p‐coumaric acid) (PPCA) for precise cysteine detection in living single‐cells, thereby overcoming cellular heterogeneity. The nanosensor reveals that the increased original intracellular cysteine production levels, particularly those exceeding 400 amol s^−1^, serve as a reliable predictor of high susceptibility to disulfidptosis. The relationship between the cell signaling pathway of cysteine metabolism with disulfidptosis is demonstrated by the nanosensor, which is further substantiated through a comprehensive analysis of diverse tumor cell lines and primary tumor cells in a mouse model. This study proposes a new indicator of disulfidptosis, and the developed nanosensor is poised to become an indispensable tool for both disulfidptosis research and the evaluation of tumor therapeutic strategies.

## Graphical abstract

Cysteine attracts interest due to its unique electrochemical activity and emerging relationship with disulfidptosis. This study develops a new electrochemical nanosensor, which represents the first of its kind capable of detecting intracellular cysteine for various types of tumor cells and primary tumor cells in mice to evaluate disulfidptosis susceptibility.

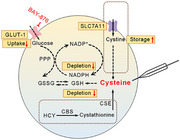

## 1 Introduction

Disulfidptosis, identified in 2023, is a novel form of programmed cell death driven by oxidative‐reductive reactions and disulfide bond formation, distinguishing it from other known cell death types [1]. Specifically, under glucose deprived conditions, cells with SLC7A11^high^ level exhibit excessive cystine accumulation [2, 3, 4]. However, due to inadequate nicotinamide adenine dinucleotide phosphate (NADPH) supply, cystine cannot be promptly reduced to cysteine, leading to abnormal accumulation of disulfides within cells [1, 5]. This disulfide stress triggers abnormal disulfide cross‐linking between actin cytoskeletal proteins, leading to cytoskeletal contraction, disrupting cytoskeletal organization, and ultimately causing the collapse of the actin network [6, 7]. Consequently, cells lose their ability to maintain normal morphology and function, ultimately leading to cell death [8, 9].

The heightened sensitivity of cells with SLC7A11^high^ level expression to disulfidptosis under glucose‐deprived conditions offers novel insights and therapeutic targets for treatment and diagnosis in cancer or other diseases [10, 11]. However, cellular heterogeneity leads to variability in the treatment responses, necessitating the development of more accurate methods and biomarkers to assess cellular susceptibility to disulfidptosis. The detection of disulfidptosis primarily involves measuring the content of disulfide compounds, the expression levels of related genes, and the changes in cell morphology [9]. Other traditional analytical methods fail to account for cellular heterogeneity, making it difficult to perform simple and efficient analyses, let alone dynamically monitor the process or evaluate the effects of related drugs. For instance, fluorescence lacks quantifiability, flow cytometry is complex and subjective, and most methods only allow verification after disulfidptosis has already occurred [12, 13, 14]. Therefore, finding a novel marker and establishment of a method to evaluate the susceptibility and process of disulfidptosis in advance is of great significance for optimizing tumor eradication strategies, facilitating drug screening, and reducing treatment costs for patients.

In cells with SLC7A11^high^ level, the excessive accumulation of cystine during glucose deprivation is a key factor leading to disulfidptosis. Nevertheless, during the practical detection procedure, it was observed that cystine exhibits an absence of electrochemical response coupled with suboptimal solubility. As documented in prior research, the transformation of cystine into cysteine has been demonstrated to enhance its solubility by over 2000‐fold. Consequently, cysteine, being the most intrinsically related compound with cystine, is selected as the focal point for electrochemical detection [15, 16]. However, current methods for cysteine detection, such as ion chromatography, gas chromatography mass spectrometry, and capillary electrophoresis, are relatively complex and demanding in terms of instrumentation, posing certain limitations [17, 18, 19]. At present, the quantification of cysteine in a single living cell in situ has not been achieved. In contrast, nanoelectrodes offer unique advantages in cytobiology research, due to their minute size, which allows for precise, deeper exploration of electrochemical behaviors without any damage to the cells [20, 21]. Owing to its favorable electrochemical activity and solubility, cysteine presents a more advantageous target than cystine for detection within cells using a nanoelectrode.

In this study, we develop a PPCA and Pt nanoparticles modified CFNE (PPCA/Pt/CFNE) nanosensor aimed at investigating the relationship between cysteine levels and disulfidptosis across different untreated cells. The incorporation of Pt nanoparticles enhances the effective area of the electrode, while PPCA induces a pore size effect and enables specific cysteine detection through hydrophobic interactions, which concentrate the analyte on the sensor surface [22]. Our sensor successfully achieves precise cysteine detection in various cells under different induction conditions. Furthermore, we demonstrate its potential for detecting cysteine in primary tumor cells derived from mice. To the best of our knowledge, this study represents the first electrochemical approach for single‐cell cysteine detection, providing a new perspective for understanding cell death mechanisms and potentially offering new strategies for innovative therapeutic treatment for cancers and other diseases.

## 2 Results and Discussion

### 2.1 Construction of the Celluar Disulfidptosis Model

**FIGURE 1:**
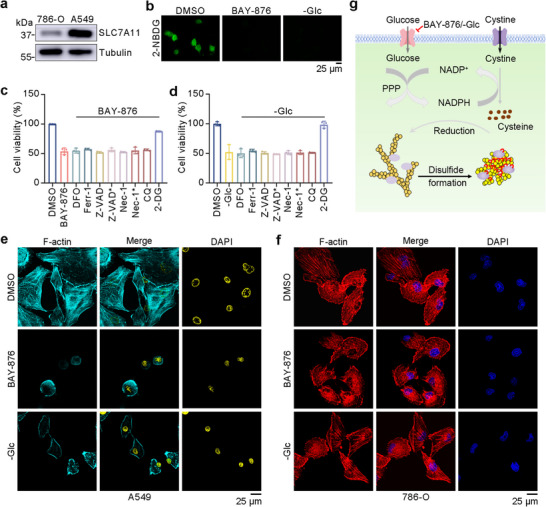
Construction and verification of the disulfidptosis cell model. (a) Western blotting analysis of SLC7A11 in A549 and 786‐O cells. (b) Glucose uptake levels in A549 cells treated with DMSO, 10 µm BAY‐876, and ‐Glc medium, incubating with 50 µm 2‐Deoxy‐2‐[(7‐nitro‐2,1,3‐benzoxadiazol‐4‐yl)amino]‐D‐glucose (2‐NBDG). (c),(d), MTT analysis in A549 cells induced by 10 µm BAY‐876 (c) and ‐Glc (d) with different death inhibitors (20 µm DFO, 10 µm Ferr‐1, 5 µm Z‐VAD, 10 µm Z‐VAD^*^, 5 µm Nec‐1, 10 µm Nec‐1^*^, 20 µm CQ, and 2 mm 2‐DG, respectively) for 10 h. Data in (c–d) were mean ± SD from 3 independent experiments. (e),(f) Fluorescent staining of F‐actin with phalloidin in A549 cells (e) and 786‐O cells (f) treated with DMSO, 10 µm BAY‐876, and ‐Glc for 10 h. Nuclei were stained with 4',6‐Diamidino‐2‐phenylindole dilactate (DAPI). (g) Mechanism of disulfidptosis in the A549 cell model.

We initiated our investigation by examining SLC7A11 expression within A549 cells and 786‐O cells, which were chosen as positive and control cell modes for inducing disulfidptosis in subsequent experiments, respectively. A notably elevated expression of SLC7A11 was revealed in A549 cells (Figure 1a). As illustrated in Figure 1b, both BAY‐876 (a GLUT‐1 inhibitor) and glucose‐free conditions (‐Glc) effectively inhibited glucose uptake, resulting in glucose deprivation. Subsequently, investigations using thiazoyl blue tetrazolium bromide (MTT) examined the effects of various cell death inhibitors on BAY‐876 and glucose‐deprivation‐induced cell death. Neither deferoxamine (ferroptosis inhibitor, DFO), ferrostatin‐1 (ferroptosis inhibitor, Ferr‐1), Z‐VAD‐fmk (apoptosis inhibitor, Z‐VAD), necrostatin‐1 (necroptosis inhibitor, Nec‐1), nor chloroquine (autophagy inhibitor, CQ) could rescue cells from BAY‐876 and ‐Glc‐induced death in A549 cells (Figure 1c,d). However, 2‐deoxyglucose (disulfidptosis inhibitor, 2‐DG) supplementation suppressed cell death manifestation. These results suggested that none of these inhibitors affects BAY‐876 and glucose‐deprivation‐induced disulfidptosis in A549 cells. Furthermore, we found prolonged induction time correlated with decreased cell survival in A549 (Figure S1a,b). However, 786‐O showed insignificant cell death, whether in the case of glucose starvation or after the addition of various cell death inhibitors (Figure S1c,d).

We then focused on analyzing actin cytoskeleton dynamics in A549 and 786‐O cells during glucose deprivation. Notably, both BAY‐876 and ‐Glc treatments induced significant F‐actin contraction in A549 cells (Figure 1e). This effect was reversed by 2‐DG treatment, which supplied NADPH through the pentose phosphate pathway, thereby preventing the inhibition of disulfidptosis (Figure S1e). Conversely, 786‐O cells displayed no remarkable morphological changes (Figure 1f). This suggests that disulfidptosis is mainly due to the abnormal accumulation of disulfide caused by glucose deficiency, which induces cell death in a way (Figure 1g). These findings collectively that the disulfidptosis cell model was successful.

### 2.2 Electrochemical Performance of the Prepared Cysteine Nanosensor

**FIGURE 2:**
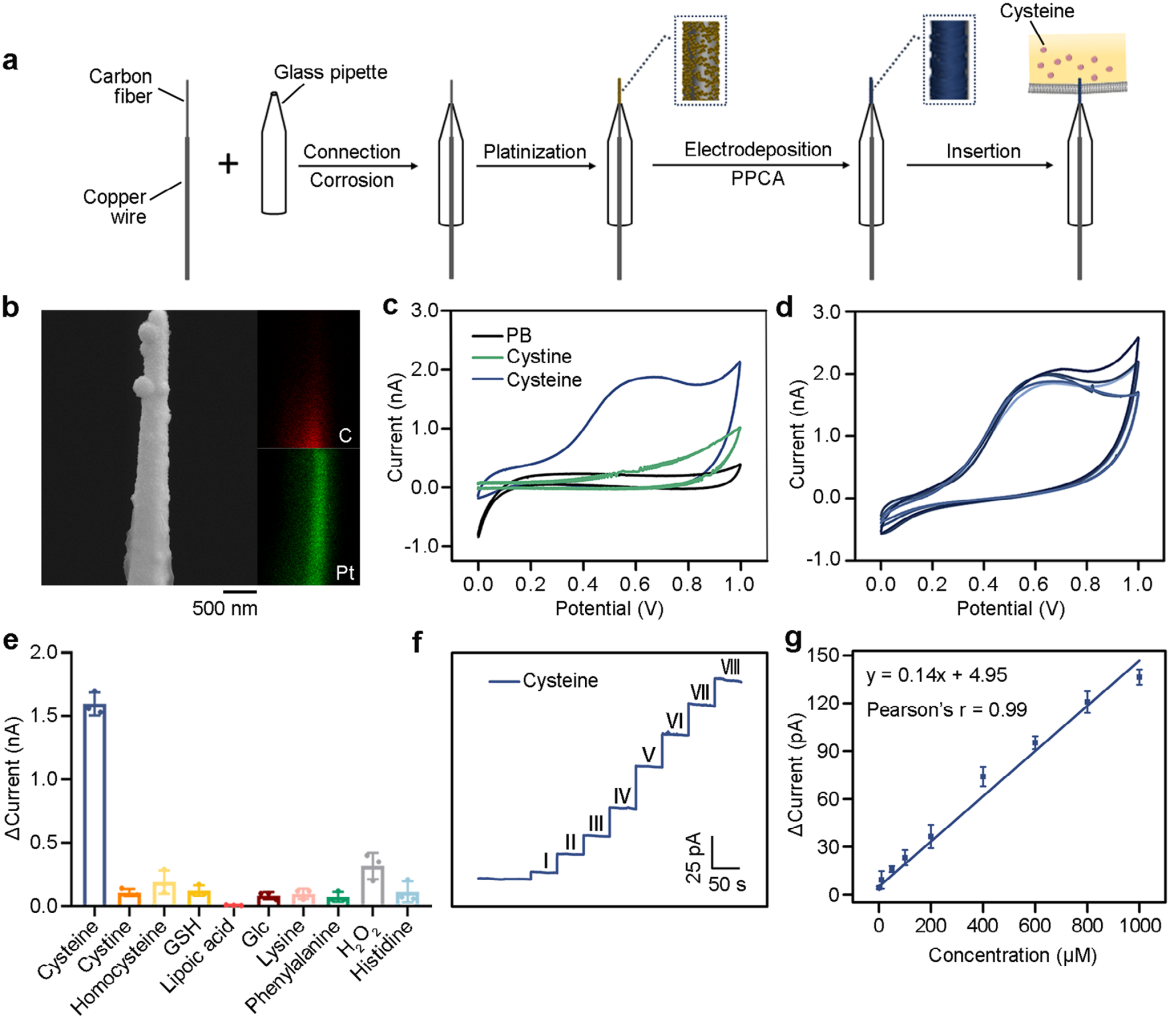
Synthesis and characterization of PPCA/Pt/CFNE. (a) Schematic illustration of the fabrication of PPCA/Pt/CFNE for cysteine detection. (b) SEM and EDX image of PPCA/Pt/CFNE. (c) CVs of cysteine (200 µm), cystine (200 µm), and PB by PPCA/Pt/CFNE. (d) Reproducibility analysis of PPCA/Pt/CFNE for cysteine detection. RSD: 4.80 %, *n* = 5. (e) Anti‐interference analysis of PPCA/Pt/CFNE for cysteine detection at 0.65 V. The concentration of cysteine was 200 µm, the concentrations of cystine, homocysteine, glutathione (GSH), lipoic acid, glucose (Glc), lysine, phenylalanine, H_2_O_2_, and histidine were 800 µm. *n* = 3. (f) Amperometric responses of PPCA/Pt/CFNE to a series of increasing cysteine concentrations at the potential of 0.65 V. (I – VIII represented 1 µm, 10 µm, 50 µm, 100 µm, 200 µm, 500 µm, 800 µm, 1000 µm, respectively.) (g) The corresponding calibration curves. *n* = 3. Data in (e and g) were mean ± SD from 3 independent experiments.

The general synthetic procedure for preparing PPCA/Pt/CFNE is illustrated in Figure 2a. Scanning electron microscopy (SEM) analysis showed that the bare CFNE exhibited a smooth surface (Figure S2a), while the Pt‐modified CFNE displayed dense and continuous nanoparticle coverage (Figure S2b). The PPCA coating appeared as a film, confirming the successful formation of a polymer porous coating on the electrode surface. SEM‐energy‐dispersive X‐ray (SEM‐EDX) analysis further confirmed the homogeneous distribution of elements within the PPCA/Pt/CFNE (Figure 2b). Electro‐polymerization results showed that the redox current continuously decreased with the increase of the number of scanning cycles, which was due to the formation of a non‐conductive polymer film leading to the obstruction of the electrode surface, and we found that five scans were sufficient for electro‐polymerization (Figure S2c). Subsequently, testing in potassium ferricyanide solution confirmed the successful construction of the PPCA/Pt/CFNE (Figure S2d).

Further amperometric responses were used to verify the ability of the PPCA/Pt/CFNE to detect cysteine, showing a notable current response to 200 µm cysteine at 0.65 V, while cystine did not show any electrical signal response similar to phosphate buffer (PB) (Figure 2c). The electrochemical responses of the five electrodes at the same concentration of cysteine were highly consistent, with a relative standard deviation of 4.80 % (Figure 2d), demonstrating excellent reproducibility of PPCA/Pt/CFNE in both preparation and performance. In addition, the PPCA/Pt/CFNE exhibited remarkable selectivity for cysteine under the conditions existed common interfering substances, especially when cystine and other amino acids are present (Figure 2e). After continuous addition of cysteine, the current response showed a linear relationship with cysteine concentration in the range of 1–1000 µm at 0.65 V (Figure 2f,g). The detection capability of PPCA/Pt/CFNE was further tested by cyclic voltammetry (CV) (Figure S2e,f). All these results confirmed that the excellent sensing performance of PPCA/Pt/CFNE in terms of quantification, dynamic response, stability, and selectivity to cysteine.

### 2.3 Quantitative Monitoring of Cysteine in Single‐Cells

**FIGURE 3:**
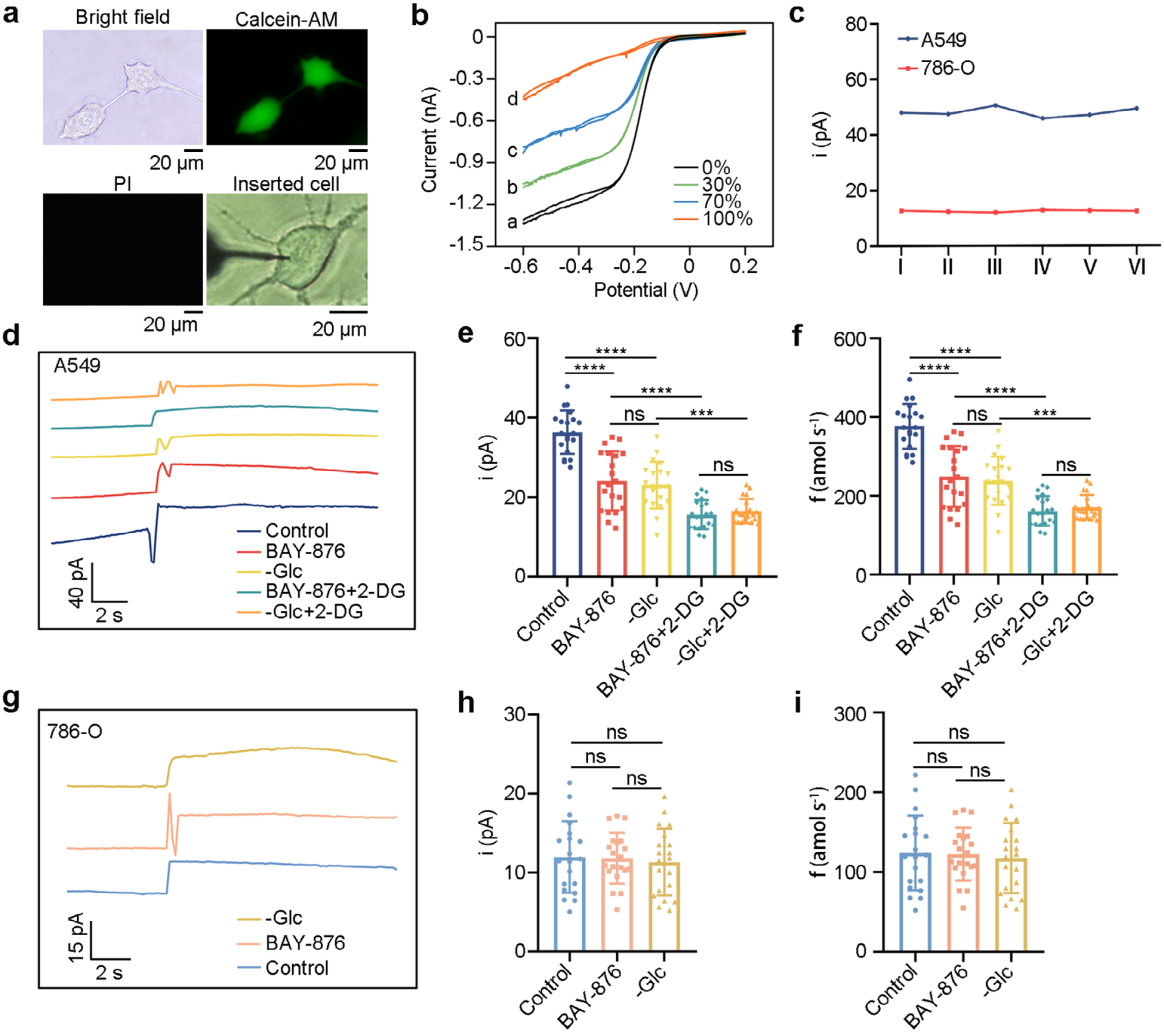
Monitoring cysteine of PPCA/Pt/CFNE insertion into cells. (a) Bright field and fluorescence imaging of A549 cells penetrated by PPCA/Pt/CFNE (indicated by arrows) after staining with calcein‐AM (green) and PI (red), and a photo of PPCA/Pt/CFNE inserted into an A549 cell. (b) CVs at different penetration depths of a PPCA/Pt/CFNE into an A549 cell when Ru(NH_3_)_6_Cl_3_ was added to the PB cell bath. (c) Current variations obtained at random positions within a single A549 and 786‐O cell. (d–f) Cysteine fluxes quantified in A549 cells by PPCA/Pt/CFNE. Amperometric responses (d) of PPCA/Pt/CFNE in PB solution with different induction conditions. Applied potential was 0.65 V. Current variations (e) and cysteine production rates (f) quantified in A549 cells with different induction conditions, *n* = 20. (g–i) Cysteine fluxes quantified in individual 786‐O cells. The amperometric responses (g) of PPCA/Pt/CFNE in PB solution with different induction conditions. Applied potential was 0.65 V. The current variation (h) and cysteine production rates (i) quantified in 786‐O cells with different induction conditions. *n* = 20. The n represented the number of randomly selected different cells for the experiment. Statistical differences: ^*^ *p* < 0.05, ^**^ *p* < 0.01, ^***^ *p* < 0.001, ^****^ *p* < 0.0001, and ns, not significant.

Motivated by its outstanding advantages, we delved deeper into exploring the potential of PPCA/Pt/CFNE for single‐cell monitoring. To confirm that the PPCA/Pt/CFNE does not cause cell damage, the PPCA/Pt/CFNE was inserted into the cells and gently pulled out, followed by fluorescence staining with calcein‐AM and propidium iodide to assess cell viability [23]. We could observe the PPCA/Pt/CFNE successfully inserted into the cells, and the results revealed no significant cell damage during the procedure (Figure 3a). Next, we further validated the PPCA/Pt/CFNE insertion mechanism by adding Ru(NH_3_)_6_Cl_3_ to the cell culture medium. Since the positively charged Ru^3+^ complex cannot penetrate intact cell membranes, the reduction current of Ru^3+^ gradually decreased as the PPCA/Pt/CFNE was inserted deeper into the cell, and the current dropped significantly after complete insertion (Figure 3b). These results confirmed that PPCA/Pt/CFNE is suitable for single‐cell detection. Furthermore, we assessed the stability of intracellular cysteine levels by electrochemically inserting electrodes into random locations in the cytoplasm of the same cells. The obtained current levels showed no significant differences with the RSD of 3.9 % for A549 cells and 3.8 % for 786‐O cells (Figure 3c), and the insertion process was shown in Movie S1. All these results indicated that the PPCA/Pt/CFNE could be effectively utilized for monitoring cysteine in single cells.

Next, we investigated the intracellular cysteine content during glucose starvation in A549 and 786‐O cells, induced by BAY‐876 and ‐Glc medium. Given the high abundance of endogenous cysteine in cells, distinct amperometric signals were immediately observed upon insertion of the PPCA/Pt/CFNE into A549 cells as the control group, and the decreased changed current were showed induced by BAY‐876 and ‐Glc (Figure 3d,e). The current signal was further converted into fluxes, and the statistical results also showed that the total of cysteine in a single A549 cell decreased from 400 to 200 amol s^−1^. This reduction may be attributed to glucose starvation slowing cysteine consumption in A549 cells. However, the adding of 2‐DG further decreased the content of cysteine in cells, and the specific mechanism needs to be further explored (Figure 3f). Furthermore, we independently conducted assessments of the cysteine levels in twenty 786‐O cells. Excitingly, these cells demonstrated remarkably low cysteine fluxes of approximately 100 amol s^−1^ and maintained stable cysteine levels even under glucose deprivation (Figure 3g–i). This stability is attributed to the low expression of SLC7A11 in 786‐O cells, which reduces the transport of cystine into the cytosol, resulting in a correspondingly low cysteine content. Consequently, these cells were less susceptible to disulfidptosis. Based on the above results, we made a preliminary speculation that cancer cells with high levels of cysteine are more sensitive to disulfidptosis induced by glucose deprivation.

### 2.4 Measuring Cysteine in Single‐Cells under Different SLC7A11 Expression

**FIGURE 4:**
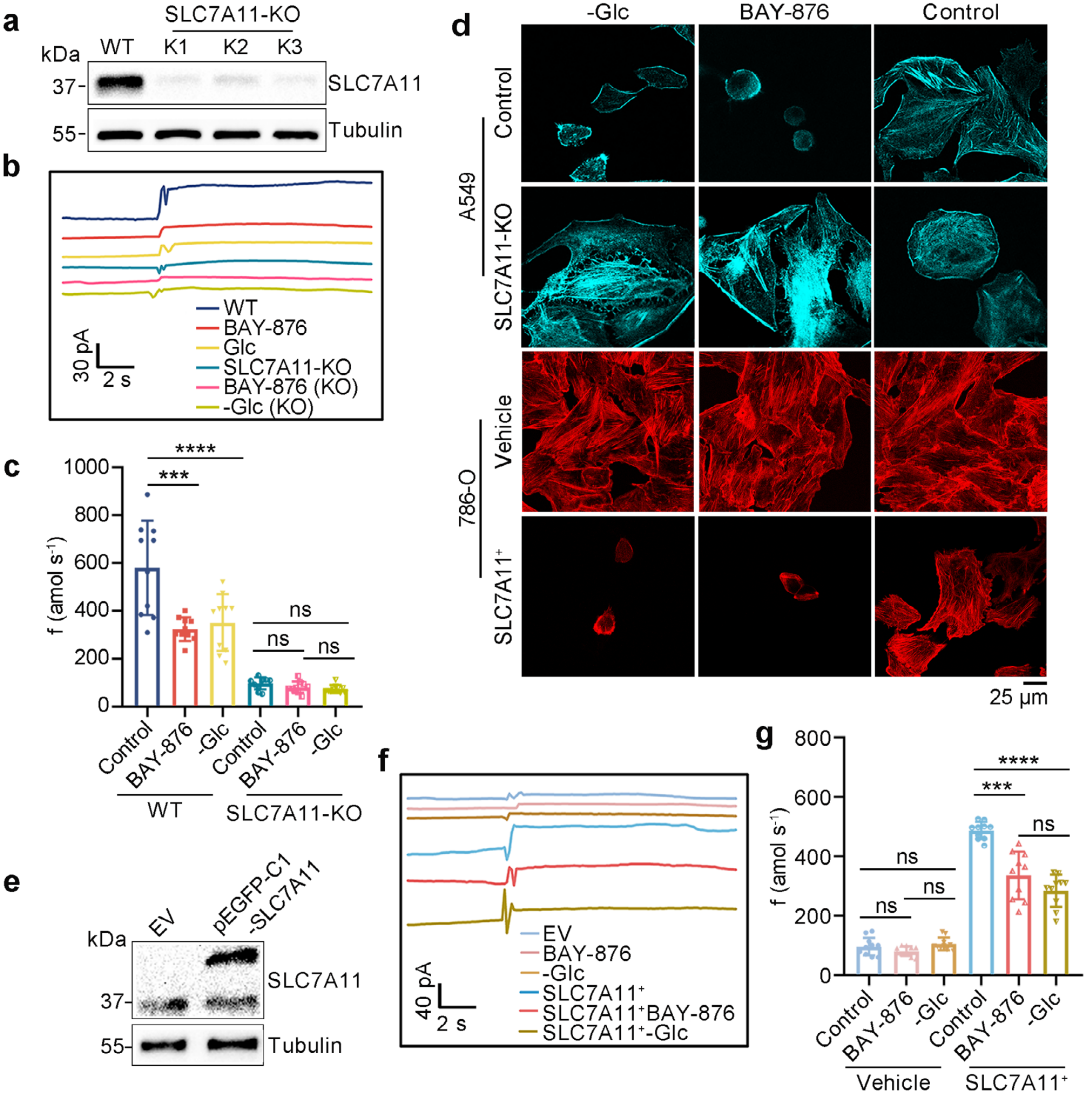
Analysis of cysteine production rates in different cells with different SLC7A11 expression. (a) Western blotting analysis with WT and SLC7A11‐KO (K1, K2, K3) in A549 cells. (b),(c) Amperometric responses (b) of PPCA/Pt/CFNE in PB solution with different induction conditions. Applied potential was 0.65 V. Cysteine fluxes (c) of PPCA/Pt/CFNE in A549 cells with different induction conditions, *n* = 10. (d) Fluorescent staining of F‐actin with SLC7A11‐KO in A549 cells and SLC7A11^+^ in 786‐O cells cultured in BAY‐876 (10 µm) and ‐Glc. (e) Western blotting analysis with empty vector (EV) and pEGFP‐C1‐SLC7A11 in 786‐O cells. (f),(g) Amperometric responses (f) of PPCA/Pt/CFNE in PB solution with different induction conditions. Applied potential was 0.65 V. Cysteine fluxes (g) of PPCA/Pt/CFNE in 786‐O cells with BAY‐876 and ‐Glc in the condition of vehicle and SLC7A11^+^, respectively. *n* = 10. The n represented the number of randomly selected different cells for the experiment. Statistical differences: ^*^ *p* < 0.05, ^**^ *p* < 0.01, ^***^ *p* < 0.001, ^****^ *p* < 0.0001, and ns, not significant.

SLC7A11, a critical reverse transporter, plays a pivotal role in cellular cystine uptake. Its high expression facilitates increased cystine intake under glucose starvation conditions, ultimately triggering disulfidptosis. To further explore the relationship between cysteine and disulfidptosis. Subsequently, we knocked out SLC7A11 in A549 cells (SLC7A11‐KO) (Figure 4a) and subjected them to glucose deprivation conditions. Next, we used the PPCA/Pt/CFNE to systematically analyze the electrochemical response characteristics of A549 cells under a variety of treatment conditions (Figure 4b). Notably, this was confirmed by the cysteine fluxes released at the cell being significantly diminished and remaining relatively stable under conditions of glucose deprivation, by around a factor of four compared with control (Figure 4c).

Concurrently, the fluorescence was used to verify the accuracy of electrochemical detection, and the experimental results demonstrated that SLC7A11‐KO prevented disulfidptosis, as evidenced by the preservation of optimal F‐actin structure in A549 cells. Similarly, staining results indicated that SLC7A11‐overexpressing (SLC7A11^+^) cells exhibited F‐actin contraction under glucose deprivation in 786‐O cells, suggesting the occurrence of disulfidptosis after transfection with SLC7A11 induced by BAY‐876 or ‐Glc (Figure 4d).

Simultaneously, we investigated the cysteine levels in 786‐O cells overexpressing SLC7A11 under various inductions (Figure 4e), and it showed an ideal transfection efficiency of about 60 % (Figure S3a,b). Concurrently, we used the constructed PPCA/Pt/CFNE to detect 10 cells under different treatment states (Figure 4f), and the experimental results showed that the production rates of cysteine in untreated 786‐O cells were generally lower than 150 amol s^−1^, and after overexpression of SLC7A11, the cysteine fluxes increased sharply approximately, approximately three times that of the control group (Figure 4g). These results all confirm that the PPCA/Pt/CFNE can perform sensitive quantitative detection of cysteine in single cells. Moreover, the higher the content of cysteine, the higher the possibility of disulfidptosis.

### 2.5 Predicting Disulfidptosis Susceptibility under Different Adding Cystine

**FIGURE 5:**
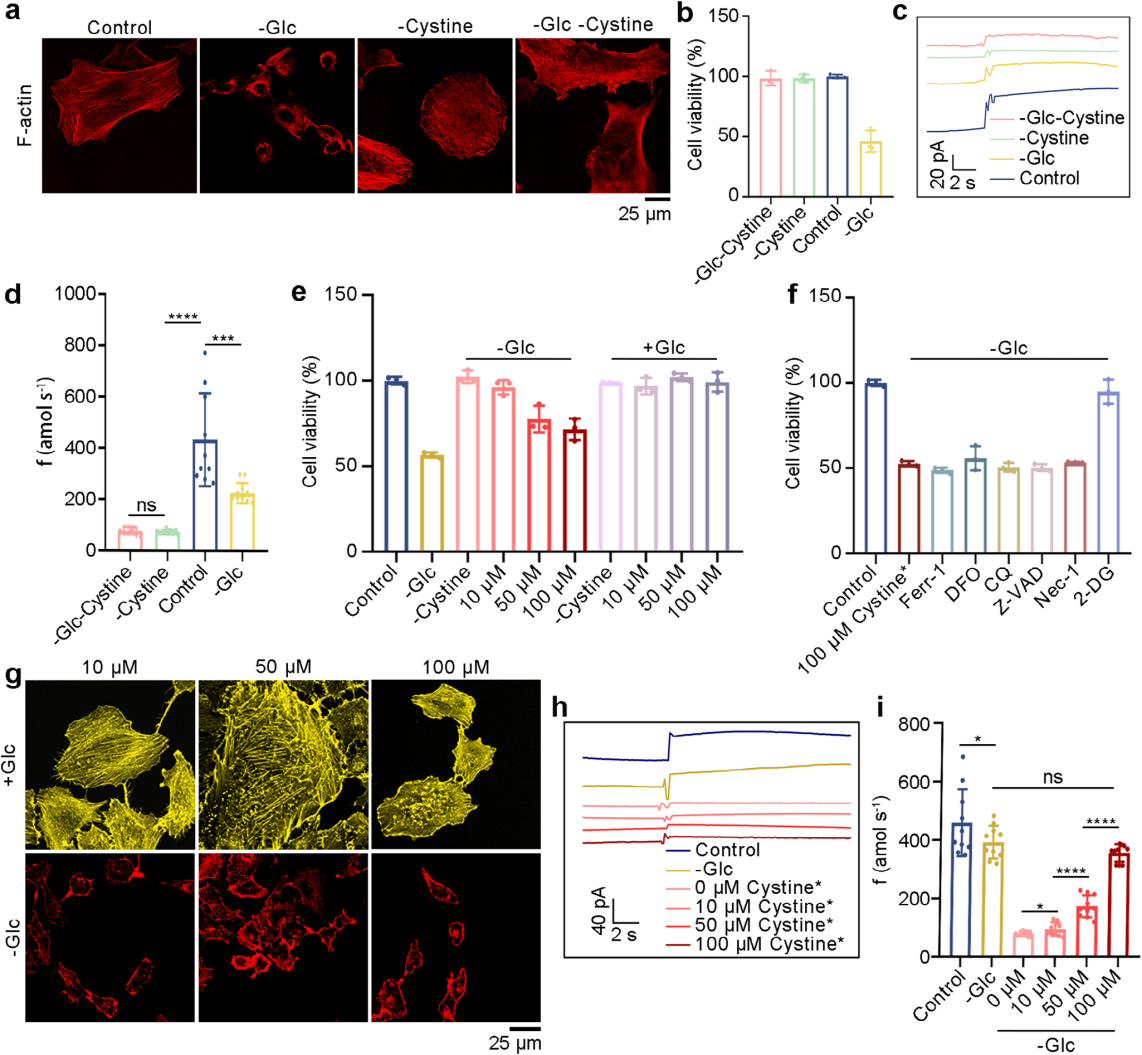
Analysis of cysteine fluxes in A549 cells with different adding cystine. Cystine^*^ represents the cystine that comes from the culture medium. (a) Fluorescent staining of F‐actin with phalloidin in A549 cells cultured in control, ‐Glc, cystine‐free (‐cystine), glucose‐ and cystine‐free (‐Glc‐cystine) medium. (b) Cell viability of A549 cells cultured in control, ‐Glc, ‐cystine, ‐Glc‐cystine medium. (c),(d), Amperometric responses (c) and cysteine production rates (d) by PPCA/Pt/CFNE in A549 cells with control, ‐Glc, ‐cystine, ‐Glc‐cystine medium, respectively. *n* = 10. (e) Cell viability of A549 cells cultured in +Glc or ‐Glc medium with different concentrations of cystine^*^. (f) Cell viability of A549 cells cultured in ‐Glc containing 100 µm cystine^*^ with indicated concentrations of other cell death inhibitors and 2‐DG (20 µm DFO, 10 µm Ferr‐1, 10 µm Z‐VAD, 10 µm Nec‐1, 20 µm CQ, and 2 mm 2‐DG, respectively). (g) Fluorescent staining of F‐actin with phalloidin in A549 cells cultured with 10 µm cystine^*^, 50 µm cystine^*^, 100 µm cystine^*^ in the condition +Glc or ‐Glc. (h),(i) Amperometric responses (h) and cysteine production rates (i) by PPCA/Pt/CFNE in A549 cells in control, ‐Glc, ‐cystine, ‐Glc 10 µm cystine^*^, ‐Glc 50 µm cystine^*^, ‐Glc 100 µm cystine^*^. *n* = 10. The n represented the number of randomly selected different cells for the experiment. Statistical differences: ^*^ *p* < 0.05, ^**^ *p* < 0.01, ^***^ *p* < 0.001, ^****^ *p* < 0.0001, and ns, not significant.

Additional evidence supports our proposed hypothesis, we found that removing cystine from A549 cell culture significantly reversed actin contraction under glucose starvation (Figure 5a) and largely prevented cell death (Figure 5b). At the same time, the electrochemical detection results showed that after the removal of cystine, the content of cysteine decreased to about 100 amol s^−1^ (Figure 5c,d). Further, we found that in A549 cells with ‐Glc, different concentrations of cystine in the medium also induced cell death, and the degree of disulfidptosis increased with the increase of cystine concentration (Figure 5e). After the addition of 2‐DG, cell survival increased, but could not be inhibited by other known cell death inhibitors (Figure 5f). Staining results showed F‐actin contraction, which may be caused by the depletion of intracellular NADPH after the addition of cystine, inducing the formation of the actin skeleton protein disulfide bond, thereby triggering disulfidptosis (Figure 5g). Finally, we used the PPCA/Pt/CFNE to monitor the change of cysteine fluxes under different induction conditions (Figure 5h,i), and the results showed that with the increase of cystine concentration, the intracellular cysteine content also increased, and disulfidptosis occurred significantly when the intracellular cysteine production rates were higher than 400 amol s^−1^.

### 2.6 Exploration on the Production Pathway of Cysteine and the Universality of PPCA/Pt/CFNE

**FIGURE 6:**
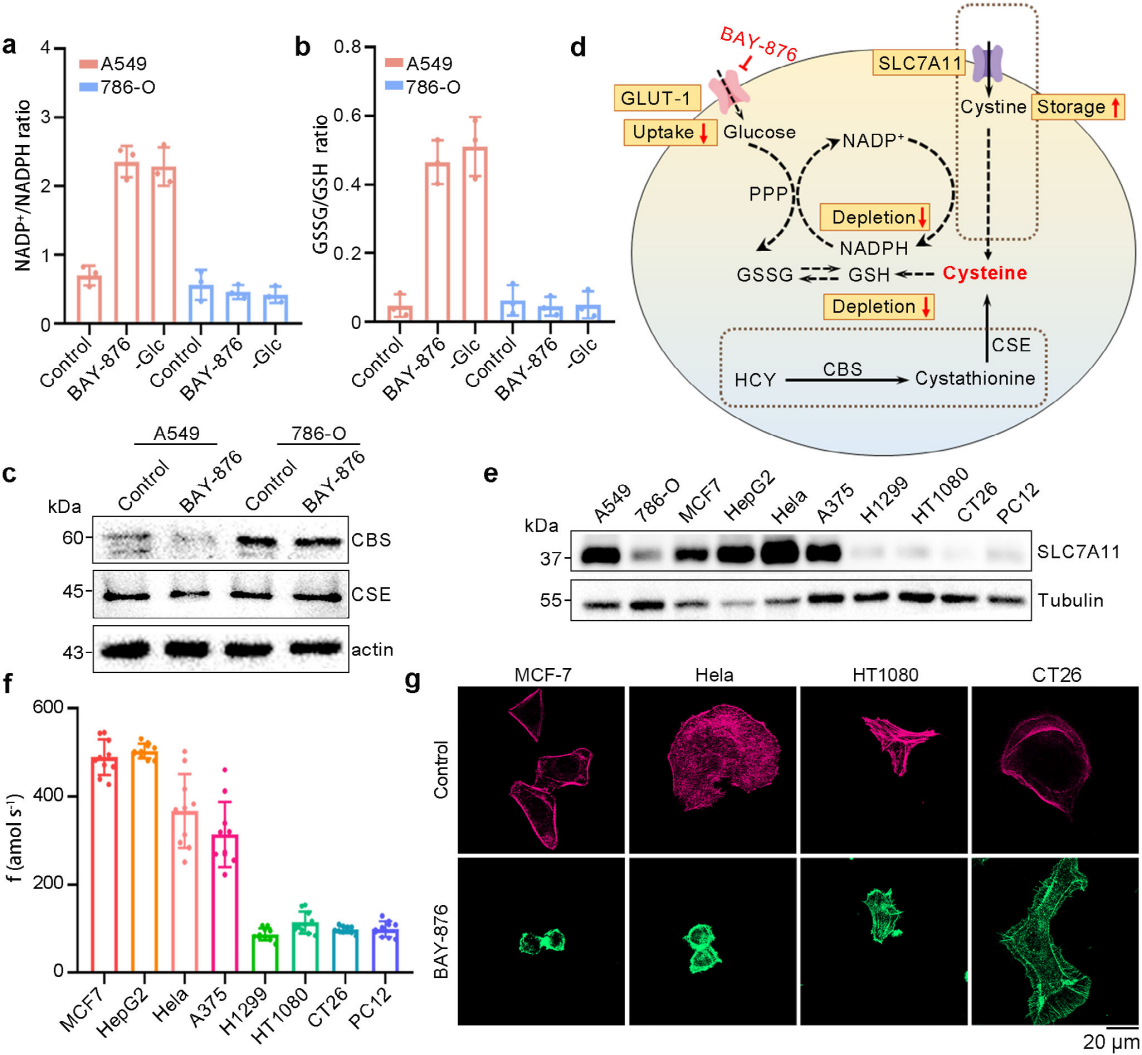
Exploration on the production pathway of cysteine and the universality of PPCA/Pt/CFNE. (a) Quantification of NADP^+^/NADPH ratios in A549 and 786‐O cells treated with DMSO, BAY‐876, and ‐Glc medium. *n*= 3. (b) Quantification of GSSG/GSH ratios in A549 and 786‐O cells treated with DMSO, BAY‐876, and ‐Glc medium. *n* = 3. Data in (a and b) were mean ± SD from 3 independent experiments. (c) Western blotting analysis of CBS and CSE in A549 and 786‐O cells treated with DMSO and BAY‐876. (d) Mechanism of disulfidptosis in the A549 cell model. (e) Western blotting analysis of SLC7A11 in different cells. (f) Cysteine production rates by PPCA/Pt/CFNE in different cells, respectively. *n* = 10. The n represented the number of randomly selected different cells for the experiment. (g) Fluorescent staining of F‐actin with phalloidin in different cells cultured with DMSO and BAY‐876.

Further notice, we delved deeply into the changes of various substances during the disulfidptosis process. The ratio of NADP^+^ to NADPH was significantly increased in A549 cells with high expression of SLC7A11 under the condition of glucose starvation, indicating NADPH depletion (Figure 6a). The ratio of GSSG to GSH showed similar trends, as demonstrated in Figure 6b. In addition to transporter‐mediated cysteine uptake, cysteine can be synthesized through the intracellular trans‐sulfurization pathway (TSP). As shown in Figure 6c, cystathionine‐beta‐synthase (the first rate‐limiting enzyme in the transsulfuration pathway, CBS), expression displayed an inverse correlation with SLC7A11 levels, which is hardly expressed in A549 cells but is highly expressed in 786‐O cells, while cystathionine gamma‐lyase (a cysteine protein sulfatase, CSE) showed no significant distinction. Moreover, there were no significant changes in the contents of CBS and CSE in the two cells before and after disulfidptosis. The total content of cysteine in A549 was higher than that of 786‐O as detected by our nanosensor. These findings demonstrated that in cells with high SLC7A11 expression, the homeostasis of cysteine primarily depends on transporter‐mediated uptake, which is a process that requires substantial NADPH to reduce cystine, as shown in Figure 6d. On the contrary, cells with low SLC7A11 expression rely on the trans‐sulfurization pathway‐derived cysteine, which does not consume NADPH. In the case of glucose deprivation, NADPH and GSH levels are depleted and in turn lead to disulfidptosis in cells with high SLC7A1 expression.

Further, we verified the expression of SLC7A11 in various cells (Figure 6e) and thereby verified the single intracellular cysteine content by our nanosensor (Figure 6f). The experimental results indicated that cancer cells with high expression levels of SLC7A11 had higher intracellular cysteine reserves and a stronger susceptibility to disulfidptosis (Figure 6g). These results demonstrated that the PPCA/Pt/CFNE can be used for intracellular cysteine detection in multiple cell lines. At the same time, it was further proved that disulfidptosis occurred significantly when the intracellular cysteine content was higher than 400 amol s^−1^.

### 2.7 Predicting Murine Tumor Therapeutic Effect Through Disulfidptosis using Nanosensors

**FIGURE 7:**
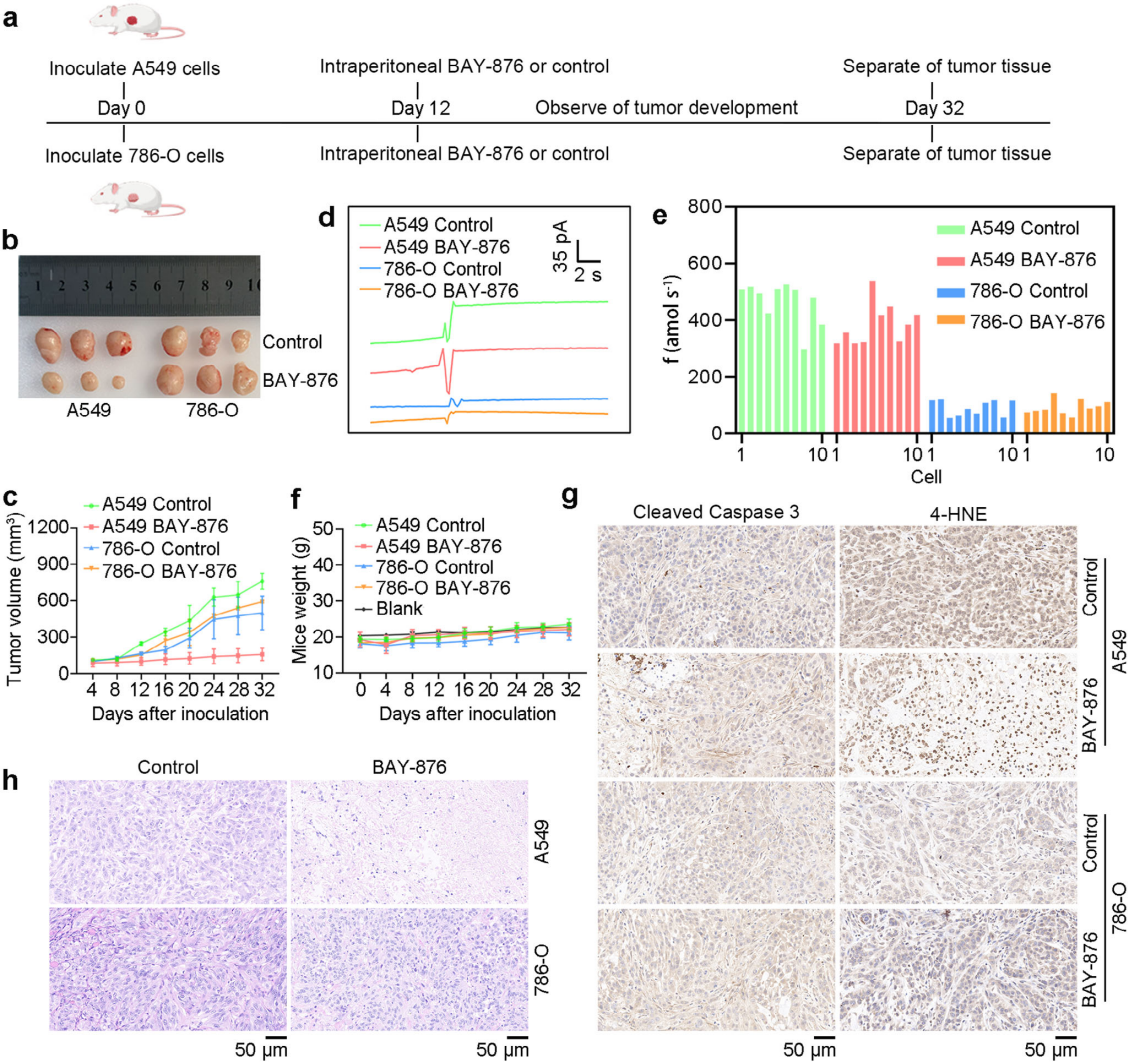
Construction and cysteine detection of disulfidptosis in mouse models. (a) Time chart of the vivo experiments. (b) Dissected tumors from NOD‐Scid by inoculating A549 and 786‐O cells. (c) Tumor volumes with indicated treatments over time. *n* = 3. (d),(e) Amperometric responses (d) and cysteine production rates (e) by PPCA/Pt/CFNE in A549 and 786‐O from the primary tumor with different induction conditions. *n* = 10. (f) Mice weights with indicated treatments over time. *n* = 3. (g) Representative immunochemical images of cleaved caspase 3 and 4‐HNE from different tumors by indicated treatments. (h) Hematoxylin and eosin stainings of different tumors with indicated treatments. Control represented 1 % DMSO, 40 % PEG 300, 5 % Tween‐80, 54 % Saline. Data in (c and f) were mean ± SD from 3 mouse.

In order to further substantiate our hypothesis, we constructed a mouse tumor model to verify the relationship between disulfidptosis and cysteine (Figure 7a), revealing that BAY‐876 treatment effectively inhibited the growth of A549 tumors, whereas no significant impact was observed on 786‐O tumors (Figure 7b,c; Figure S4a). Here, when the tumor grew to an appropriate size, we first removed a small number of tumor cells for primary culture and conducted electrochemical detection. The experimental results showed that the cysteine content in A549 cells was significantly higher than that in 786‐O cells, and the cysteine content in A549 cells decreased slightly after treatment (Figure 7d,e). So, we conclude that disulfidptosis is more likely to occur in cells with cysteine content higher than 400 amol s^−1^.

Soon afterward, BAY‐876 treatment neither caused alterations in animal body weight and elicited notable pathological changes in major organs (Figure 7f; Figure S4b), nor did it affect the staining patterns of cleaved caspase‐3 (an apoptosis marker) or 4‐Hydroxynonenal (a lipid peroxidation marker, 4‐HNE), apoptosis and ferroptosis were ruled out (Figure 7g). The tumor tissue treated with BAY‐876 showed an obvious death phenomenon by inoculated A549 cells (Figure 7h). This provided further confirmation that the diminution in tumor size observed in mice, which had been injected with A549 cells, subsequent to BAY‐876 treatment, was attributable to the manifestation of disulfidptosis. All aforementioned findings converge on a conclusion that disulfidptosis was more likely to occur in cells with cysteine fluxes higher than 400 amol s^−1^.

## 3 Conclusions

Disulfidptosis as a novel form of programmed cell death contributes to a deeper understanding of the complex mechanisms of cell death and demonstrates significant potential implications in the field of targeted tumor therapy [24]. At present, the research on disulfidptosis is still in the preliminary development stage, and the detection methods are relatively limited [3]. Cysteine, an important medium in the process of disulfidptosis, is very crucial to monitor in real time [14].

In this study, we developed a PPCA/Pt/CFNE capable of specific detection of cysteine in living single cells, enabling the assessment of the probability of disulfidptosis in different normal cancer cells by monitoring intracellular cysteine levels. The results indicated a positive correlation between the disulfidptosis susceptibility and cysteine content in unprocessed cancer cells. Moreover, our study speculated that when the intracellular cysteine production rates exceeded 400 amol s^−1^, disulfidptosis was more prone to occur, which was further validated in mouse models and different cell lines.

This disulfidptosis phenomenon may be attributable to the fact that for the vast majority of cancer cells, they mainly rely on SLC7A11 to transport cystine and further convert it into cysteine owing to the TSP seems not very active. Therefore, cells with SLC7A11^high^ level tend to promote the accumulation of cystine and subsequently increase the level of cysteine, and increase the possibility of disulfidptosis. Our electrochemical nanosensors confirmed the credibility of this result.

Our findings provided a more accurate, convenient, and fast method for predicting disulfidptosis susceptibility and offered fresh perspectives and methodologies for the treatment of cancer and related diseases. In addition, our researches underscore the potential of nanosensors as a robust platform for conducting in‐depth studies on cysteine‐related cellular metabolism.

## 4 Experimental Section

### 4.1 Cell Experiments

A549 (Research Resource Identifier (RRID)): CVCL_0023), 786‐O (RRID: CVCL_1051), MCF7 (RRID: CVCL_0031), HepG2 (RRID: CVCL_0027), Hela (RRID: CVCL_0030), A375 (RRID: CVCL_0132), H1299 (RRID: CVCL_0060), HT1080 (RRID: CVCL_0317), CT26 (RRID: CVCL_7254), and PC12 (RRID: CVCL_0481) cell lines were purchased from the cell bank of the Chinese Academy of Sciences (China). All the cellular lines were contamination‐free (tested by the vendor). A549, MCF7, HepG2, Hela, A375, HT1080, and PC12 cells cultured in Dulbecco's Modified Eagle medium (DMEM) supplemented with 10 % fetal bovine serum (FBS), and 786‐O, H1299, and CT26 cells were cultured in RPMI1640 medium with 10 % FBS. All the cells were grown in a humidified atmosphere with 5 % CO_2_ and 37 °C. The ‐Glc DMEM, ‐Glc RPMI1640, and ‐Glc‐cystine DMEM were specially customized from Procell (China). Then, medium with‐cystine was prepared by adding cystine to the Glc medium. When glucose deprivation was performed, cells were cultured with 10 µm BAY‐876 (MedChemExpress, China) or ‐Glc medium for 10 h.

### 4.2 Fabrication of PPCA/Pt/CFNE

To fabricate CFNE, the carbon fiber microelectrode, which was prepared as reported previously, was held on the edge of a butane flame to get the right size [25]. The carbon fiber (Good Fellow, UK) and copper wires were first connected together, then inserted into a borosilicate capillary (World Precision Instruments, USA) and etched to the desired size with a flame. The CFNE was inserted into the 0.5 mm H_2_PtCl_6_ solution for ensuring a suitable black‐platinum by 3‐electrode circuit with CV (‐0.5 – 0 V, 100 mV.s^−1^). Then PPCA film was electropolymerized on the face of the electrode from potentiodynamic electrolysis containing 0.1 m NaOH by applying CV (0 – 1 V, 25 mV·s^−1^ for 5 scans) to fabricate one PPCA/Pt/CFNE.

### 4.3 Electrochemical Data Acquisition and Analysis

All electrochemical tests involving cells were carried out on shockproof tables and faraday cages. Cells were placed under an inverted microscope (40x objective), and the PPCA/Pt/CFNE was moved near the cells by a micromanipulator and carefully inserted into the cells. All the amperometric responses were recorded by a CHI630E electrochemical analyzer (Shanghai Chenhua Instrument Co., LTD., China).

To verify the accurate insertion of the nanoelectrode into cells, 2 mm Ru(NH_3_)_6_Cl_3_ was added to the PB and utilized as an electrochemical probe due to its impermeability through the cell membrane. The current yielded the correct result that the reduction current of Ru^3+^ progressively decreased with continuous electrode insertion. The current was recorded at a constant potential of 0.65 V after multiple washed the cell with PB.

### 4.4 Quantitative Characterization of Individual Cysteine Fluxes

I_species_ (t) represents the individual limiting plateau current of species that will be concurrently observed at time t [20]. Based on Faraday's law, the time‐dependent production rate of cysteine, denoted as f_species_ (t), can subsequently be calculated from the temporal change in the individual current, represented as i_species_ (t) [23, 26]. i_species_ (t) = Fnf, where F, with a value of 9,6500 C/mol, denotes the Faraday constant, and n_species_ stands for the electron stoichiometry associated with the electrochemical oxidation of the species at the PPCA/Pt/CFNE (n_cysteine_ = 1) [22].

### 4.5 Cell Viability Detection

The 5 × 10^3^ – 1 × 10^4^ cells were seeded into 96‐well plates and incubated overnight. After the cells were treated with special drugs, 20 µL of MTT solution of 5 mg mL^−1^ was added to each well cultured in the incubator for 4 h. Subsequently, 200 µL of DMSO was added to each well, and the absorbance at 490 nm was determined using a spectrophotometer.

To prove that the nanoelectrode was nearly nondestructive to the cells, PPCA/Pt/CFNE were inserted into cells and removed to withdraw. Calcein‐AM and PI (final concentration of 3 µg mL^−1^) were then added to the medium. After incubation under cell culture conditions for 15 min, the residual dye was washed with PB (three times), and the bright‐field and fluorescence micrographs were recorded by an inverted fluorescence microscope.

### 4.6 Different SLC7A11 Expression in Different Cells

For A549, spread the cells evenly into the six‐well plate to ensure the cells reached 60 % when transfected. The final concentration of siRNA was 20 µm by adding RNase‐free H_2_O. Following the instructions provided in the kit, a liposome‐siRNA complex was formed and then added to a 6‐well plate, where it was incubated for 6 h. Subsequently, the 1mL complete medium was added and cultured for 48 h with 5 % CO_2_ at 37 °C. All operations were performed in an RNA‐free environment.

For 786‐O, the cells were evenly spread on the 6‐well plate to ensure that the cells reached 60 % when transfected. Mix 100 µL opti‐MEM and 2 µg DNA to incubate for 6 h, then add 1 mL complete medium, 5 % CO_2_, and incubate at 37 °C for 48 h.

### 4.7 Fluorescent Staining

Glucose uptake experiments were conducted according to the methods as described earlier [27]. A549 cells were treated with the ‐Glc and BAY‐876 at 37 °C with 5 % CO_2_ and with a ‐Glc medium containing 50 µm 2‐[N‐(7‐nitrobenz‐2‐oxa‐1,3‐diazol‐4‐yl) amino]‐2‐deoxy‐d‐glucose (Amgicam, China), washed cells with PB, and used an inverted fluorescence microscope for image.

About the fluorescent staining of F‐actin, modified according to the previous experimental scheme [28]. Briefly, the cells adhering to the slides were rinsed twice with PB and subsequently fixed with 4 % paraformaldehyde at ambient temperature for 20 min. Permeate with 0.1 % Triton X‐100 in PB for 10 min, followed by another PB washing. Subsequently, the cells were incubated in actin‐stain 555 phalloidin (Beyotime Biotechnology, China) (1:50 dilution) in the dark at room temperature for 30 min. Cell nuclei were stained with DAPI. Finally, all fluorescence images were captured using a focal microscope (Leica, TCS SP8 MP).

### 4.8 Western Blotting Analysis

The β‐Tubulin Monoclonal Antibody was purchased from Beyotime Biotechnology. The β‐actin Monoclonal Antibody was purchased from Proteintech. SLC7A11 monoclonal antibody was supplied by Abcom. The CBS polyclonal antibody was bought from Sangon Biotech. The CSE antibody was obtained from Abways.

On the basis of previous experiments, appropriate optimization has been carried out. The whole‐cell extracts were individually resolved on SDS‐polyacrylamide gel electrophoresis (SDS‐PAGE) gels and then transferred to polyvinylidene fluoride membranes. Then blocked using 5 % skim milk powder for 2 h. Subsequently, they were incubated with primary antibodies diluted powder at 4 °C for an overnight period. Following this, the membranes were thoroughly washed with Tris‐buffered saline containing 0.1 % Tween‐20 five times to remove any excess primary antibodies. Finally, the membranes were incubated with horseradish peroxidase‐conjugated secondary antibodies for 1 h at room temperature. Following additional washes with Tris‐buffered saline containing 0.1 % Tween‐20, the membranes were subsequently visualized through the application of an intensified chemiluminescent detection reagent.

### 4.9 Animal Experiments

This research complied with all relevant ethical regulations of Shandong Normal University, including the Institutional Review Board and Institutional Animal Care and Use Committee. The ethical approval number was AEECSDNU2025080. The 4–6 weeks female mice (NOD‐Scid) were purchased from Syagen Biotechnology Co., LTD. (China), and raised in pathogen‐free conditions with free access to water and food at 25±2 °C. After 7 days, 1 × 10^6^ cells (786‐O) or 5 × 10^5^ cells (A549) were resuspended in 100 µL PB and injected into the subcutaneous tissue of mice. Tumor growth was monitored in all mice using 2D tumor measurements. The tumor volume was calculated as volume = 0.5 × length × width^2^. When the tumor volume reached about 50 – 100 mm^3^, each group of mice (*n* = 5) was randomly divided into 4 groups, intraperitoneal injection of 3 mg kg^−1^ BAY‐876 or control (1 % DMSO, 40 % PEG 300, 5 % Tween‐80, 54 % Saline) by intraperitoneal administration every 2 days. The tumor burden did not exceed the 1.5 cm limit allowed by the Ethics Committee.

To obtain primary tumor cells, fresh mouse tumor tissues were sterilized and moved to a super clean table and washed repeatedly with PB to guarantee the cleanliness of the epidermis and the absence of any impurities. Subsequently, the tissue was meticulously dissected into the smallest possible fragments and transferred to a centrifugal tube containing collagenase 4, placed on a shaking table at 37 °C and agitated at 220 rpm for a duration of 5 – 6 h. The mixture was then passed through a 70 µm sieve for filtration. Centrifuge the filtered liquid at 1500 rpm for 10 min. After centrifugation, the supernatant was removed, and the bottom precipitation was retained. Then, the bottom precipitation in the prepared primary cell medium was re‐suspended, centrifuged at 1500 rpm for 10 min after re‐suspension, the supernatant was removed, and the primary cell medium was employed to resuspend the cells. Finally, moved them to a place in a large petridish. Follow‐up experiments could be carried out once the cells adhered to the dish surface.

### 4.10 Statistical Analysis

There was no use of a statistical method to predetermine the sample size, no exclusion of data from the analyses, and all analyses were conducted without randomization, thereby ensuring the utmost reproducibility. Statistical analyses were performed using GraphPad Prism 8.0.1 and OriginPro 2016. All data were presented as the mean ± standard deviation (SD). Differences between two groups were assessed by Student's *t*‐tests, and statistical significance was expressed as follows: ^*^ *p* < 0.05, ^**^ *p* < 0.01, ^***^ *p* < 0.001, ^****^ *p* < 0.0001, ns: not significant. Detailed statistical methods and sample size for each experiment are provided in the corresponding figure legends.

## Author Contributions

X.D. and Z.Z conceived the project and designed the experiment. C.Z., X.D., Z.Z., and J.Z. wrote and revised the paper. C.Z., X.Z., and Y.L. cultured the cell lines and constructed a cell model. C.Z., X.Z., and S.L. built the nanosensor and conducted the electrochemical experiments. X.D., Z.Z., M.J., and L.L. designed and built the single‐cell detection platform. C.Z., B.Y., M.Z., S.Z., and S.L. conducted and analyzed western blot and fluorescent staining experiments. X.D., Z.Z., J.Z., M.J and L.L. designed, built, and maintained tracking and segmentation software. C.Z., Z.M., S.Z., and F.Y. conducted and collected the data of mice models.

## Conflicts of Interest

The authors declare no conflicts of interest.

## Supporting information




**Supporting File 1**: advs73477‐sup‐0001‐SuppMat.docx.

**Supporting File 2**: advs73477‐sup‐0002‐MovieS1.mp4.

## Data Availability

The data that support the findings of this study are available from the corresponding author upon reasonable request.
