# Supplementary material for: Quantification of Single‐Cell Cysteine Using an Electrochemical Nanosensor for Predicting Tumor Disulfidptosis Susceptibility
Source: Adv Sci (Weinh). 2025 Dec 22;13(13):e23478. doi: 10.1002/advs.202523478 (PMC12955924; doi:10.1002/advs.202523478)
Supplement: Supplementary file 1 — Supporting File 1: advs73477‐sup‐0001‐SuppMat.docx. [file ADVS-13-e23478-s002.docx]

Supporting Information

Quantification of Single-cell Cysteine Using an Electrochemical Nanosensor for Predicting Tumor Disulfidptosis Susceptibility


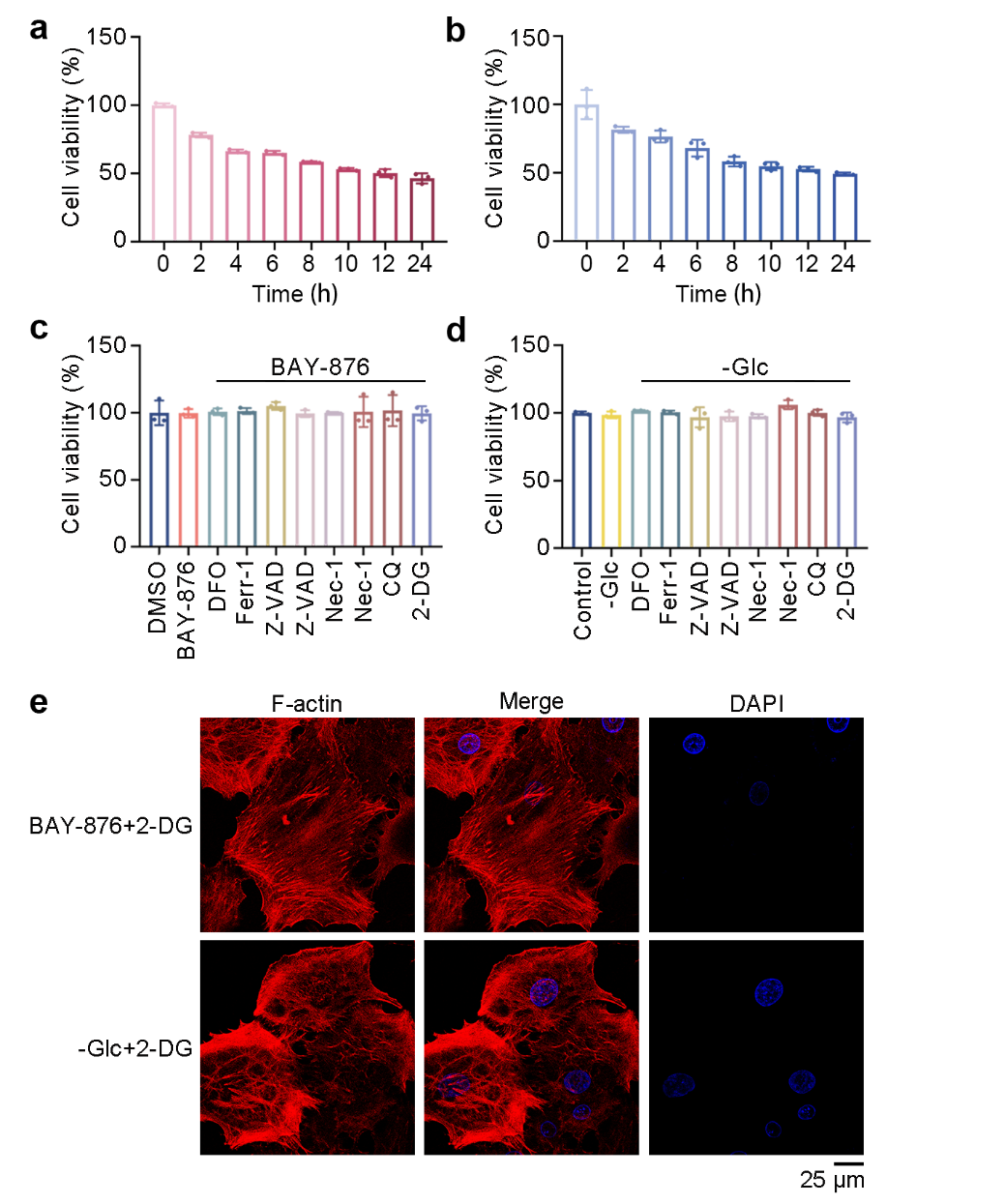
Congcong Zhang, Xiangdi Zhang, Shushen Li, Yuyang Li, Bei Yuan, Mingshuang Zheng, Shuo Zhang, Fangping Yuan, Min Jia, Lixia Lu, Jun Zhou, Zhenguo Zhang^*^, Xin Du^*^

**Figure S1. Construction and verification of disulfidptosis cell model.** **a-b,** MTT analysis in A549 cells with 8 μM BAY-876 **(a)** and -Glc **(b)** for different hours. n = 3. **c-d,** MTT analysis in 786-O cells with 8 μM BAY-876 **(c)** and -Glc **(d)** with or without co-treatment in different death inhibitors (20 μM DFO, 10 μM Ferr-1, 5 μM Z-VAD, 10 μM Z-VAD, 5 μM Nec-1, 10 μM Nec-1, 20 μM CQ and 2 mM 2-DG, respectively) for 10 h. n = 3. **e,** Fluorescent staining of F-actin with phalloidin in A549 cells treated with 8 μM BAY-876+2-DG and -Glc+2-DG. Nuclei were stained by DAPI. Data in (a-d) were mean ± SD from 3 independent experiments.

**
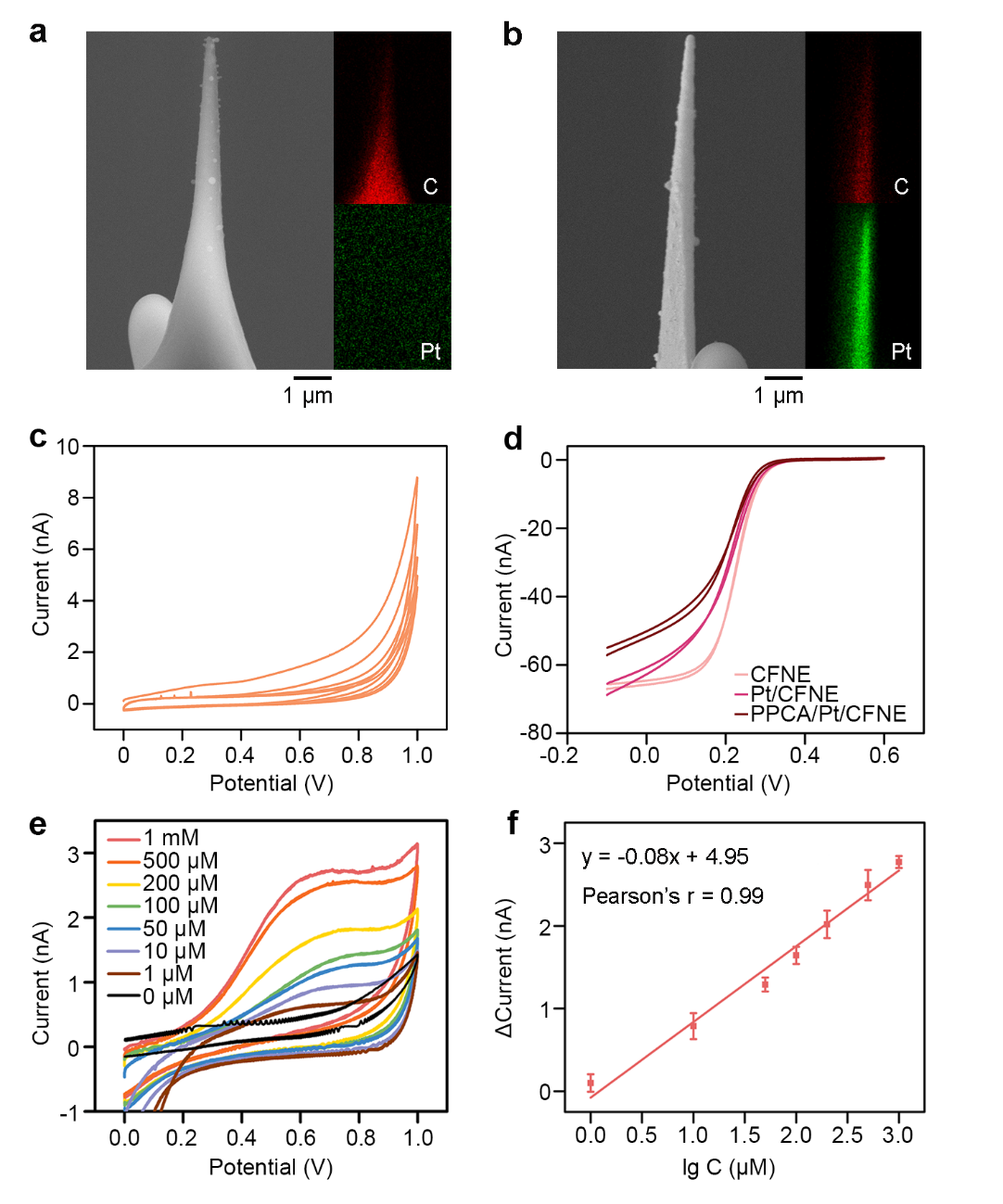
F****igure S2. Synthesis and characterization of PPCA/Pt/CFNE.** **a-b,** SEM and EDX image of CFNE **(a)** and Pt/CFNE **(b)**. **c,** Electropolymerization of 0.1 mM PPCA on Pt/CFNE in 0.1 M NaOH. Potential scan rate is 25 mV s^-1^. **d,** Verification of the CFNE, Pt/CFNE, and PPCA/Pt/CFNE by CV scanning in potassium ferricyanide. **e,** Amperometric responses of PPCA/Pt/CFNE in a series of increasing cysteine concentrations dissolved with PB at the potential of 0.65 V. Potential scan rate is 25 mV s^-1^. **f,** The corresponding calibration curves. n = 3. Data in (f) were mean ± SD from 3 independent experiments.


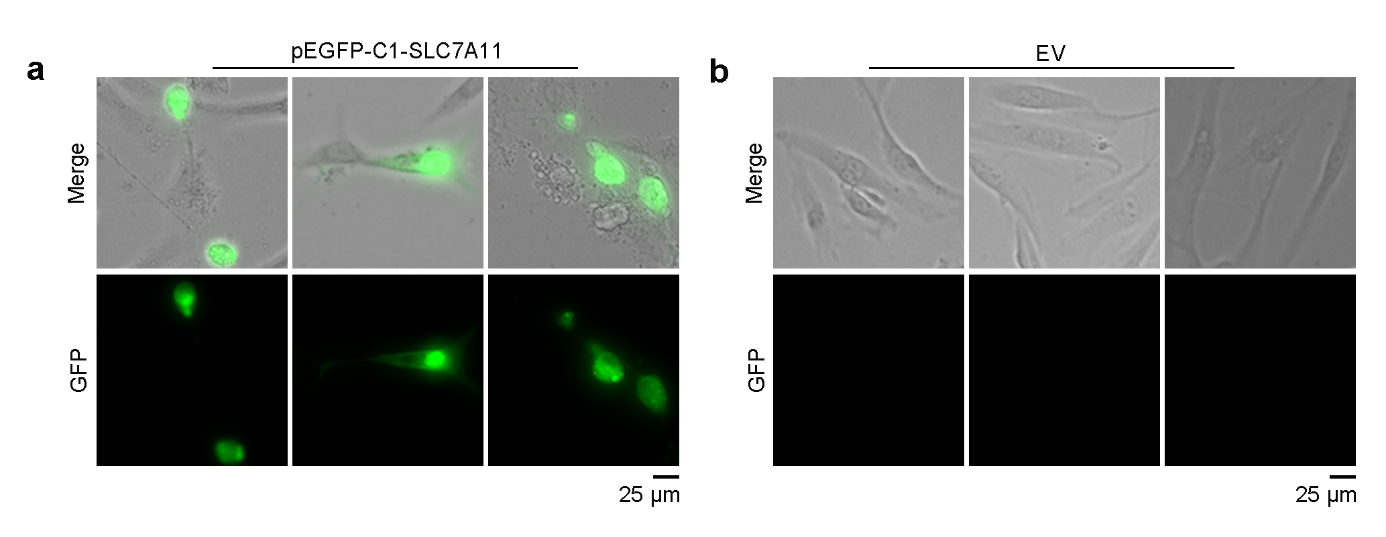


**F****igure S3. The verification of transfection efficiency of 786-O cells.** Fluorescent staining of 786-O cells transfected with pEGFP-C1-SLC7A11 **(a)** and EV **(b)**.


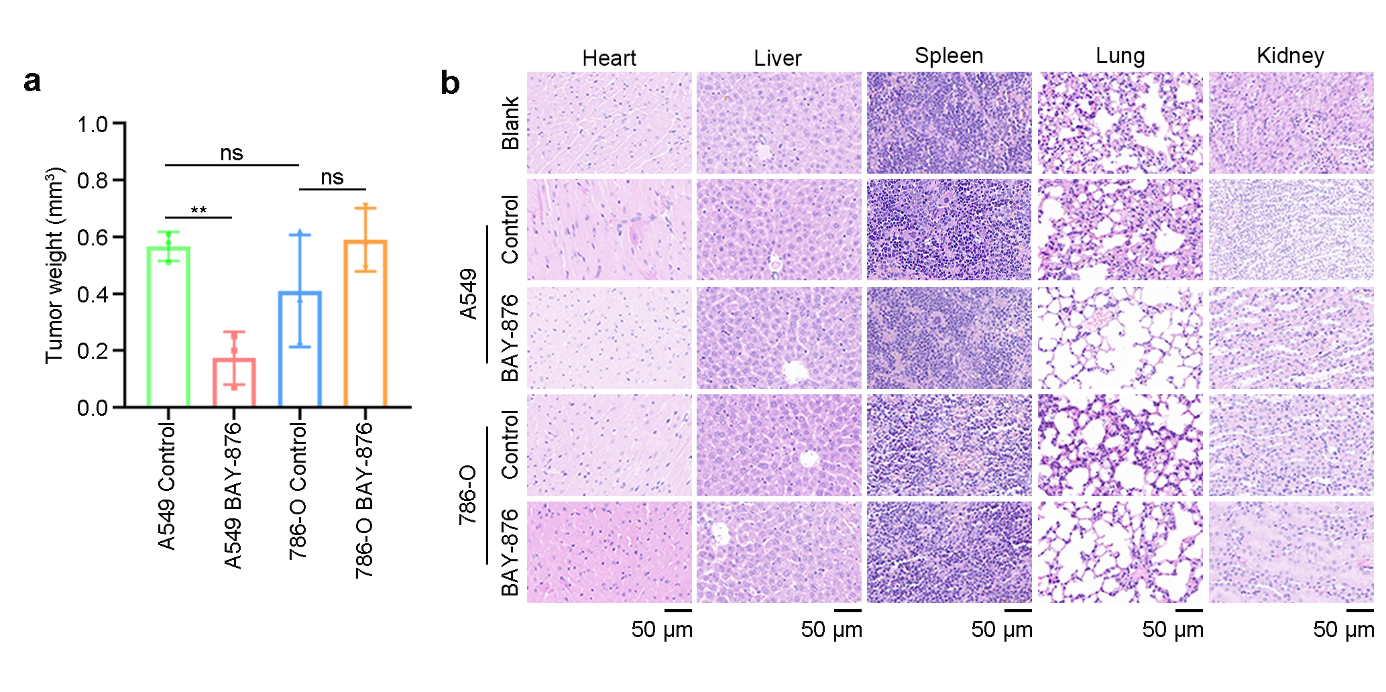


**Figure S4. The construction of disulfidptosis in mouse models.** **a,** The tumor weight statistics with different treatment. n = 3. **b,** Representative hematoxylin and eosin staining of major organs from mice treated with control or BAY-876 after inoculated A549 and 786-O cells. Data in (a) were mean ± SD from 3 mouse. Statistical differences: ^*^ *p* < 0.05, ^**^ *p* < 0.01, ^***^ *p* < 0.001, ^****^ *p* < 0.0001, and ns, not significant.
